# The Orphan Receptor GPR35 Contributes to Angiotensin II–Induced Hypertension and Cardiac Dysfunction in Mice

**DOI:** 10.1093/ajh/hpy073

**Published:** 2018-05-31

**Authors:** Nina Divorty, Graeme Milligan, Delyth Graham, Stuart A Nicklin

**Affiliations:** 1 Centre for Translational Pharmacology, Institute of Molecular, Cell, and Systems Biology, College of Medical, Veterinary and Life Sciences, University of Glasgow, Glasgow, United Kingdom; 2 Institute of Cardiovascular and Medical Sciences, College of Medical, Veterinary and Life Sciences, University of Glasgow, Glasgow, United Kingdom

**Keywords:** blood pressure, GPCR, GPR35, heart failure, hypertension, orphan receptor

## Abstract

**BACKGROUND:**

The orphan receptor G protein–coupled receptor 35 (GPR35) has been associated with a range of diseases, including cancer, inflammatory bowel disease, diabetes, hypertension, and heart failure. To assess the potential for GPR35 as a therapeutic target in cardiovascular disease, this study investigated the cardiovascular phenotype of a GPR35 knockout mouse under both basal conditions and following pathophysiological stimulation.

**METHODS:**

Blood pressure was monitored in male wild-type and GPR35 knockout mice over 7–14 days using implantable telemetry. Cardiac function and dimensions were assessed using echocardiography, and cardiomyocyte morphology evaluated histologically. Two weeks of angiotensin II (Ang II) infusion was used to investigate the effects of GPR35 deficiency under pathophysiological conditions. *Gpr35* messenger RNA expression in cardiovascular tissues was assessed using quantitative polymerase chain reaction.

**RESULTS:**

There were no significant differences in blood pressure, cardiac function, or cardiomyocyte morphology in GPR35 knockout mice compared with wild-type mice. Following Ang II infusion, GPR35 knockout mice were protected from significant increases in systolic, diastolic, and mean arterial blood pressure or impaired left ventricular systolic function, in contrast to wild-type mice. There were no significant differences in *Gpr35* messenger RNA expression in heart, kidney, and aorta following Ang II infusion in wild-type mice.

**CONCLUSIONS:**

Although GPR35 does not appear to influence basal cardiovascular regulation, these findings demonstrate that it plays an important pathological role in the development of Ang II–induced hypertension and impaired cardiac function. This suggests that GPR35 is a potential novel drug target for therapeutic intervention in hypertension.

G protein–coupled receptor 35 (GPR35) is a poorly characterized orphan G protein-coupled receptor (GPCR), which has potential therapeutic value based on its association with several diseases, including cancer, inflammatory bowel disease, diabetes, hypertension, and heart failure.^1^ Despite reports linking it to several endogenous molecules, including kynurenic acid^2^ and the chemokine CXCL17,^3^ its endogenous ligand and biological function remain undefined.^4^

Screening has identified a range of high-potency surrogate ligands for GPR35, enabling studies into its pharmacology and function.^5–8^ Although 2 antagonists were discovered several years ago,^9,10^ these are highly selective for the human orthologue^11^ and so it has been challenging to conduct robust *in vivo* studies. Nonetheless, several studies have highlighted potential roles for GPR35 in the cardiovascular system and in cardiovascular disease.

A genome-wide association study that aimed to identify novel risk genes for coronary artery disease in a population of hypertensive individuals identified an association between a *GPR35* single-nucleotide polymorphism and coronary artery calcification, a risk factor in coronary artery disease.^12^ GPR35 has also recently been implicated as a susceptibility gene for chemotherapy-induced cardiotoxicity.^13^ Further genetic evidence for effects of GPR35 in the heart was observed in 12 patients with chronic heart failure, where *GPR35* expression was found to be upregulated in myocardial tissue of these patients relative to healthy controls.^14^ In the same study, adenoviral vector-mediated overexpression of GPR35 in primary rat cardiomyocytes resulted in hypertrophy.^14^ Furthermore, a significant 37.5 mm Hg increase in systolic blood pressure was reported in GPR35 knockout mice compared with the wild-type background strain.^14^ These data strongly suggest that GPR35 plays an important role in blood pressure regulation. In neonatal mouse cardiomyocytes, both *Gpr35* messenger RNA and cell-surface protein levels were reported to increase in response to hypoxia and hypoxia-inducible factor 1 activation.^15^*Gpr35* expression was induced in the myocardium of experimental mouse models of myocardial infarction and pathological hypertrophy, and preceded cardiac remodeling and heart failure. Furthermore, *Gpr35* overexpression in mouse cardiomyocytes led to membrane ruffling and formation of retraction fibers, with no apparent change in cell size.^15^ However, the lack of GPR35 antagonists with efficacy at the rodent orthologues has hindered a more detailed understanding of these findings in rodent *in vivo* models.

*GPR35* gene expression has been detected in human primary vascular smooth muscle cells and endothelial cells from saphenous vein, and GPR35 agonists induced actin fiber reorganization and migration in vascular smooth muscle cells.^16^ These effects were blocked by the specific GPR35 antagonists CID-2745687 and ML-145 and by inhibitors of Ras homolog gene family, member A (RhoA) signaling pathway components, indicating that endogenous GPR35 mediates its effects in vascular cells through coupling to Gα_13_, as previously described in GPR35-overexpressing HEK293 cells.^17^ This suggests a potential role for GPR35 in the regulation of vascular remodeling, which occurs in intimal hyperplasia, restenosis, and vein graft failure.

The emerging roles of GPR35 in the cardiovascular system may implicate it as a novel contributor to cardiovascular dysfunction and a potential therapeutic target in cardiovascular disease, especially in hypertension and heart failure.^1^ Although GPR35 has been linked with cardiovascular disease, and a GPR35 knockout mouse has been reported to have an abnormal blood pressure phenotype, the consequences of GPR35 deficiency on cardiovascular function are yet to be examined thoroughly. Therefore, we investigated the cardiovascular phenotype of a GPR35 knockout mouse in detail under basal physiological conditions and following pathological stimulation *via* angiotensin II (Ang II) infusion.

## METHODS

### Experimental animals

Animals were housed under 12-hour light/dark cycles (0700–1900 light, 1900–0700 dark) at ambient temperature and maintained on normal chow (Special Diet Services), with drinking water provided *ad libitum*. All animals used for experimental procedures were adult, male, F2 homozygous littermates (C57BL/6 or GPR35 KO; a gift from Novartis). Genotypes of F2 offspring were determined by end-point polymerase chain reaction using primers: *Gpr35* forward: 5′ ATCGCATGCACCAGTGGACAGAGAC 3′; *Neo* forward: 5′ GACGAGTTCTTCTGAGGGGATCGATC 3′; *Gpr35* reverse: 5′ GGTCCACAGCAATGGCAGTGACCAG 3′. All procedures were conducted in accordance with the Animals (Scientific Procedures) Act 1986 under project license 60/4286, held by Dr. Delyth Graham.

Animals were randomly allocated to treatment groups (*n* = 8 for wild-type vs. knockout study and *n* = 6 for Ang II infusion model), and the experimenter was blinded to treatments during the study and analysis. If telemetry readings were outside of the physiological blood pressure range (due to technical error such as displacement of the probe) at any time during data collection, the entire data set for that animal was excluded prior to unblinding.

### Blood pressure monitoring by telemetry

PhysioTel PA-C10 pressure transmitters (DSI, St Paul, MN) were surgically implanted into 12- to 13-week-old mice, with the transmitter positioned subcutaneously on the right shoulder and the catheter inserted into the left carotid artery, as previously described.^18^ Transmitters were calibrated to verify accuracy to within 3 mm Hg before implantation. Recovery surgery was performed under aseptic conditions, and animals were anaesthetized with 2.5% isoflurane and 1.5 l/min O_2_ throughout the procedure. Following implantation, animals were administered with analgesic (5 mg/kg carprofen), recovered in a heated incubation box until fully conscious and monitored daily by weighing to assess postoperative health. Following a 7-day recovery period, transmitters were remotely switched on and measurements were recorded every 5 minutes for the duration of the study using the Dataquest IV Telemetry System (DSI). These measurements were used to calculate 24-hour means and daytime and nighttime means based on the 12-hour light/dark cycle.

### Echocardiography

Cardiac function was assessed using M-mode echocardiography, as previously described.^19^ Animals were anaesthetized with 1 l/min 1.5% isoflurane and 1.5 l/min O_2_ throughout the procedure. Images were obtained using a Siemens Acuson Sequoia 512 ultrasound unit with an 18LS probe set at a frequency of 14 MHz. Left ventricular (LV) dimensions during systole and diastole were measured using ImageJ software. Measurements were taken from 9 separate cardiac cycles over 3 different images. Fractional shortening (FS), end systolic volume, end diastolic volume, and ejection fraction (EF) were calculated using the following equations:

$$\begin{array}{lll} \text{FS }\left(\%\right) & = & ((\text{LVEDD}–\text{LVESD})/\text{LVEDD})\times \text{1}00\;\\ \text{ESV }\left(\text{ml}\right) & = & \left(\text{7}/\left(\text{2}.\text{4}+\text{LVESD}\right)\right)\times {\text{LVESD}}^{\text{3}}\\ \text{EDV }\left(\text{ml}\right) & = & \left(\text{7}/\left(\text{2}.\text{4}+\text{LVEDD}\right)\right)\times {\text{LVEDD}}^{\text{3}}\\ \text{EF }\left(\%\right) & = & ((\text{EDV}–\text{ESV})/\text{EDV})\times \text{1}00 \end{array}$$

Where LVEDD = LV end diastolic dimension (cm) and LVESD = LV end systolic dimension (cm). End systolic volume and end diastolic volume were derived using the Teichholz method.^20^

### Subcutaneous infusion of Ang II

Ang II was administered by subcutaneous infusion using ALZET micro-osmotic pumps (Model 1002; Charles River, Wilmington, MA). Infusion commenced at 13–14 weeks of age (7 days after telemetry implantation) and continued for 2 weeks until sacrifice. Ang II human (Sigma-Aldrich, Gillingham, United Kingdom) was dissolved in water and sterilized through a 0.2 µm filter. Pumps were filled with the appropriate concentration of Ang II to deliver a dose of 400 ng/kg/min (24 µg/kg/h)^21^ at a flow rate of 0.25 µl/h. After filling, pumps were primed in sterile saline overnight at 37 °C, then subcutaneously implanted in the left flank (the opposite side to the telemetry probe) under aseptic conditions, followed by administration of analgesic (5 mg/kg carprofen).

### Histological assessment of cardiomyocyte diameter

Wheat germ agglutinin was used to stain cardiomyocyte cell membranes. Tissues were fixed in 10% formalin at sacrifice. Following deparaffinization and dehydration, 5 µm heart (left ventricle + septum) sections were washed in running tap water for 5 minutes. Antigen retrieval was performed by heating with 10 mM sodium citrate solution (pH 6) with 0.1% (v/v) Tween 20 for 10 min, then sections were cooled to room temperature before blocking in 1% (v/v) goat serum + 5% (w/v) BSA for 1 hour at room temperature. Sections were then incubated with 10 µg/ml wheat germ agglutinin conjugated to Alexa Fluor 555 (Thermo Fisher Scientific, Waltham, MA) for 1 hour at room temperature protected from light. Sections were washed for 2 × 5 minutes in PBS, then mounted with ProLong Gold anti-fade reagent with DAPI (Thermo Fisher Scientific) and dried overnight at room temperature. DAPI and Alexa Fluor 555 images were taken with a ×40 objective on an Olympus BX40 microscope using a QImaging QICAM Fast 1394 camera. The experimenter was blinded to treatment groups during imaging and analysis. Cardiomyocyte width (the longest axis of a transversely orientated cardiomyocyte) was measured using ImageJ software. Approximately 50 cells from 4 fields of view were measured per animal, using a grid system to minimize bias.

### Gene expression analysis

Tissues were snap-frozen in liquid nitrogen at sacrifice and stored at −80 °C. To lyse heart and kidney tissue, 30–50 mg of tissue was transferred to an RNase-free microcentrifuge tube with 700 µl QIAzol (QIAGEN, Hilden, Germany) and two 3 mm tungsten carbide beads. Tissues were homogenized for 2 × 1 minutes in a QIAGEN TissueLyser. To lyse aorta tissue, tissue was cooled in liquid nitrogen and crushed to a fine powder using a mortar and pestle cooled on dry ice, then lysed by the addition of QIAGEN RTL buffer containing 1% (v/v) β mercaptoethanol and homogenized using a QIAshredder spin column (QIAGEN). RNA extraction was performed immediately following lysis using the miRNeasy Mini Kit (QIAGEN) (heart and kidney) or the RNeasy Fibrous Tissue Mini Kit (QIAGEN) (aorta). One microgram of RNA was reverse transcribed to complementary DNA using TaqMan Reverse Transcription Reagents (Life Technologies). qRT-polymerase chain reaction was performed using Applied Biosystems TaqMan Gene Expression Assays for *Gpr35* (Mm01973686_s1), Angiotensin receptor type 1a (*Agtr1a*) (Mm0061671_m1), and *Ppib* (Mm00478295_m1) (Life Technologies). Plates were read on a QuantStudio Flex Real-Time PCR System (Thermo Fisher Scientific) and comparative cycle threshold (C_T_) values were obtained using QuantStudio Flex software (Thermo Fisher Scientific). ΔC_T_ values were calculated (ΔC_T_ = C_T_ for *Ppib* − C_T_ for *Gpr35*). These were analyzed using the 2^−ΔΔCT^ method,^22^ in which ΔC_T_ values are compared with those of an appropriate control to generate ΔΔC_T_ values and relative quantification (RQ) values (RQ = 2^−ΔΔCT^). SEs are expressed as RQ_min_ (RQ_min_ = 2^−(ΔΔCT + SEM)^) and RQ_max_ (RQ_max_ = 2^−(ΔΔCT − SEM)^).

### Statistical analysis

Statistical analyses were carried out using Graphpad Prism software. Normal data distribution was confirmed using Shapiro–Wilk test, and data were therefore compared using 2-tailed unpaired Student’s *t* test. Hemodynamic measurements made by telemetry were compared using 2-way repeated measures analysis of variance (basal GPR35 knockout study) or Student’s *t* test on the slope of the linear regression calculated for individual animals (Ang II infusion study).

## RESULTS

### Blood pressure and cardiac function in the GPR35 knockout mouse under physiological conditions

**Figure 1.**
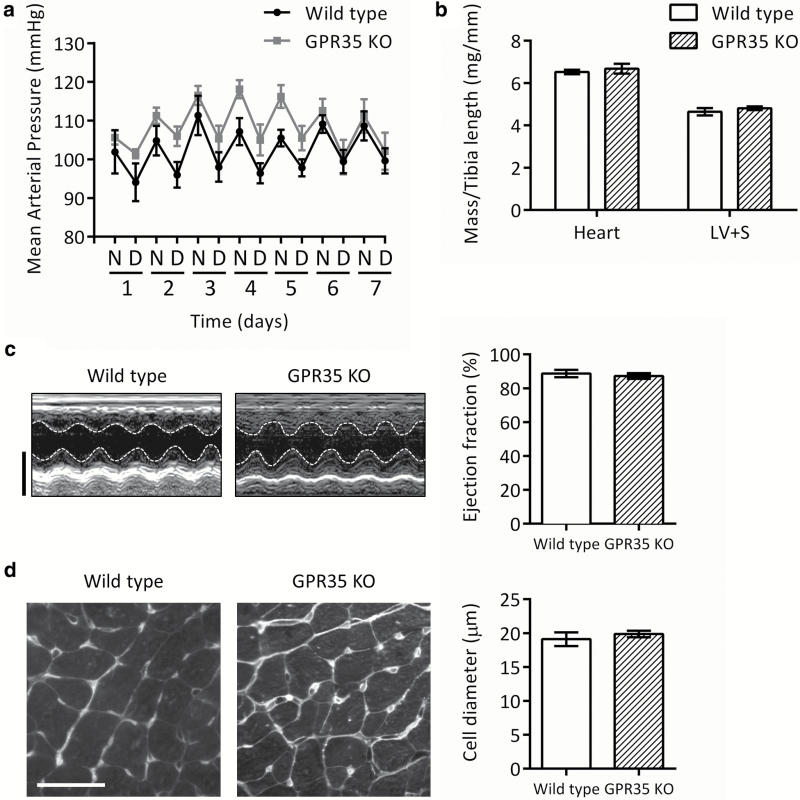
Mean arterial pressure, cardiac mass and cardiomyocyte morphology in GPR35 KO mice under physiological conditions. (**a**) Mean arterial pressure over 7 days in wild-type (black circles) and GPR35 KO (gray squares) mice. Data are 12-hour means representing night (N) and day (D) periods; *n* = 6 (wild type) or *n* = 8 (GPR35 KO). (**b**) Whole heart and LV + S mass determined at sacrifice and normalized to tibia length; *n* = 8. (**c**) Representative M-mode echocardiography images taken on the longitudinal axis at the level of the papillary muscle (scale bar = 3 mm) and ejection fraction calculated from LV dimensions; *n* = 5 (wild type) or *n* = 8 (GPR35 KO). (**d**) Transverse heart sections stained with fluorescently tagged wheat germ agglutinin (scale bar = 30 µm) and cell diameter quantified as the width of cardiomyocytes orientated on the short axis; *n* = 5. Data are mean ± SEM, compared using 2-way repeated measures analysis of variance (a) or 2-tailed unpaired *t* test (b–d); no significant differences were found. Abbreviations: GPR35 KO, G protein–coupled receptor 35 knockout; LV, left ventricular; S, septum.

Systolic, diastolic, and mean arterial blood pressure (MAP) were not significantly altered in GPR35 knockout mice vs. the wild-type strain over a 7-day period (*P* = 0.127; mean MAP 102 ± 3 and 108 ± 3 mm Hg, respectively; Figure 1a and Table 1). LV dimensions, FS, and EF were not significantly altered in GPR35 knockout mice vs. wild type (Figure 1c and Table 2). Cardiac mass and cardiomyocyte morphology were not significantly altered in GPR35 knockout mice vs. wild type (Figure 1b and d).

**Table 1.** Blood pressure in wild-type and GPR35 knockout mice under physiological conditions

Measurement	Wild type	GPR35 KO	*P* value
Systolic blood pressure (mean over 7 days), mm Hg			
Overall	112 ± 3	117 ± 3	0.201
Daytime	109 ± 3	111 ± 3	0.242
Nighttime	117 ± 3	122 ± 2	0.178
Diastolic blood pressure (mean over 7 days), mm Hg			
Overall	93 ± 3	101 ± 3	0.116
Daytime	88 ± 3	97 ± 4	0.153
Nighttime	97 ± 3	105 ± 3	0.086
Mean arterial pressure (mean over 7 days), mm Hg			
Overall	102 ± 3	108 ± 3	0.127
Daytime	97 ± 3	104 ± 3	0.165
Nighttime	107 ± 3	113 ± 2	0.101

Data are mean ± SEM of *n* = 6 (wild type) or *n* = 8 (GPR35 KO), compared using 2-tailed repeated measures analysis of variance (wild type vs. GPR35 KO); no significant differences were found. Abbreviation: GPR35 KO, G protein–coupled receptor 35 knockout.

**Table 2.** Echocardiographic parameters in wild-type and GPR35 knockout mice under physiological conditions

Dimension	Wild type	GPR35 KO	*P* value
LV wall thickness, mm	0.84 ± 0.13	0.87 ± 0.05	0.807
LV systolic diameter, mm	1.43 ± 0.14	1.53 ± 0.08	0.513
LV diastolic diameter, mm	3.06 ± 0.10	3.12 ± 0.06	0.629
Fractional shortening, %	53.5 ± 3.1	51.3 ± 1.8	0.540
End systolic volume, µl	8.98 ± 2.68	10.30 ± 1.37	0.637
End diastolic volume, µl	75.23 ± 7.48	78.91 ± 4.67	0.667
Ejection fraction, %	88.7 ± 2.2	87.2 ± 1.3	0.549

Data are mean ± SEM of *n* = 5 (wild type) or *n* = 8 (GPR35 KO), compared using 2-tailed unpaired *t* test (wild type vs. GPR35 KO); no significant differences were found. Abbreviations: GPR35 KO, G protein–coupled receptor 35 knockout; LV, left ventricular.

### Blood pressure and cardiac function in the GPR35 knockout mouse following Ang II infusion

**Figure 2.**
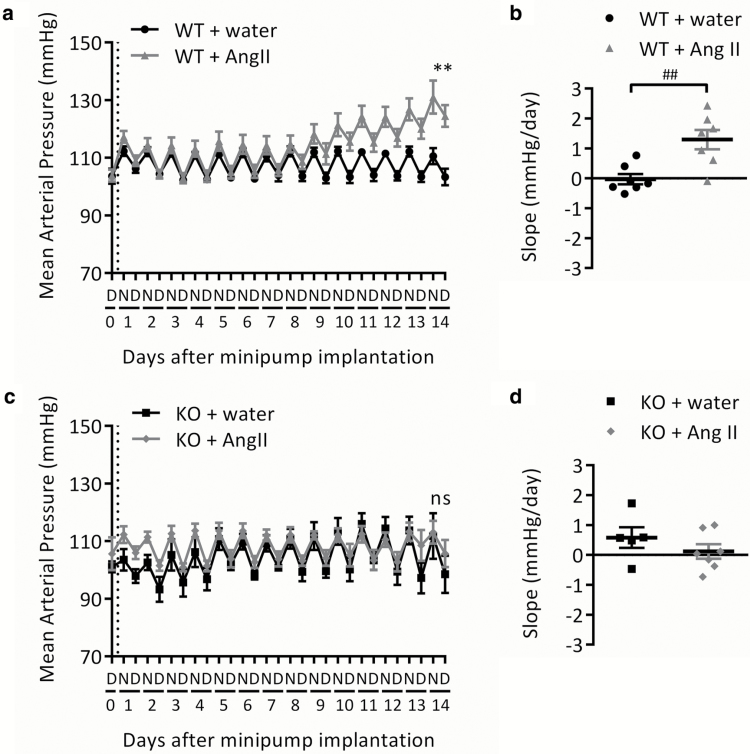
Change in mean arterial blood pressure in wild-type and GPR35 KO mice during 2-week Ang II infusion. Mean arterial blood pressure in (**a,b**) WT or (**c,d**) GPR35 KO mice. Baseline measurements were taken on day 0 prior to minipump implantation (indicated by dotted line). (a,c) 12-hour means representing night (N) and day (D) periods. (b,d) Change over 14 days presented as slope of the linear regression (mm Hg/day) for each individual animal. Data are mean ± SEM of *n* = 7 (except GPR35 KO + water, *n* = 5). **P < 0.01, compared using 2-tailed unpaired *t* test (water vs. Ang II on day 14). ^##^*P* < 0.01, compared using 2-tailed unpaired *t* test (water vs. Ang II). Abbreviations: Ang II, angiotensin II; GPR35, G protein–coupled receptor 35; KO, knockout; ns, not significant; WT, wild type.

In wild-type mice, Ang II infusion induced a steady increase in MAP of 17 mm Hg over the 2-week period (Figure 2a and Table 3). Mean arterial pressure at day 14 and the slope of the change over time were both significantly increased for wild-type mice infused with Ang II compared with those infused with water, with a difference in MAP at day 14 of 21.1 ± 4.7 mm Hg (*P* < 0.001) and a mean increase of 1.29 ± 0.33 mm Hg/day (*P* < 0.01; Figure 2a and b and Table 3). Systolic and diastolic blood pressure followed the same pattern (Table 3).

In GPR35 knockout mice, no Ang II–induced increases in MAP, systolic, or diastolic blood pressure were observed (Figure 2c and d and Table 3). There were no significant differences between Ang II- and water-infused mice in MAP at day 14 or the slope of the change over time, with a difference in MAP at day 14 of 7.2 ± 7.8 mm Hg (*P* = 0.376) and a mean slope of 0.12 ± 0.24 mm Hg/day (*P* = 0.638; Figure 2c and d and Table 3).

**Table 3.** Blood pressure in wild-type and GPR35 knockout mice following 2-week Ang II infusion

Group	Day 0	Day 14	Day 14, *P* value	Slope, mm Hg/day	Slope, *P* value
Systolic blood pressure, mm Hg					
Wild type + water	123 ± 3	120 ± 3	0.002**	−0.13 ± 0.20	0.004**
Wild type + Ang II	125 ± 1	145 ± 5		1.42 ± 0.39	
GPR35 KO + water	114 ± 2	133 ± 7	0.289	0.39 ± 0.35	0.672
GPR35 KO + Ang II	121 ± 4	123 ± 5		0.16 ± 0.36	
Diastolic blood pressure, mm Hg					
Wild type + water	94 ± 1	95 ± 4	0.015*	0.11 ± 0.15	0.003**
Wild type + Ang II	97 ± 1	112 ± 5		1.24 ± 0.27	
GPR35 KO + water	93 ± 2	99 ± 7	0.758	0.79 ± 0.34	0.195
GPR35 KO + Ang II	97 ± 4	97 ± 4		0.22 ± 0.25	
Mean arterial pressure, mm Hg					
Wild type + water	108 ± 2	107 ± 3	0.002**	−0.03 ± 0.17	0.004**
Wild type + Ang II	111 ± 1	128 ± 5		1.29 ± 0.33	
GPR35 KO + water	102 ± 2	105 ± 7	0.588	0.58 ± 0.35	0.283
GPR35 KO + Ang II	109 ± 3	109 ± 4		0.12 ± 0.24	

Data are mean ± SEM of *n* = 7 (except GPR35 KO + water, *n* = 5). *P < 0.05, **P < 0.01, compared using 2-tailed unpaired *t* test (water vs. Ang II). Abbreviations: Ang II, angiotensin II; GPR35, G protein–coupled receptor 35; KO, knockout.

**Figure 3.**
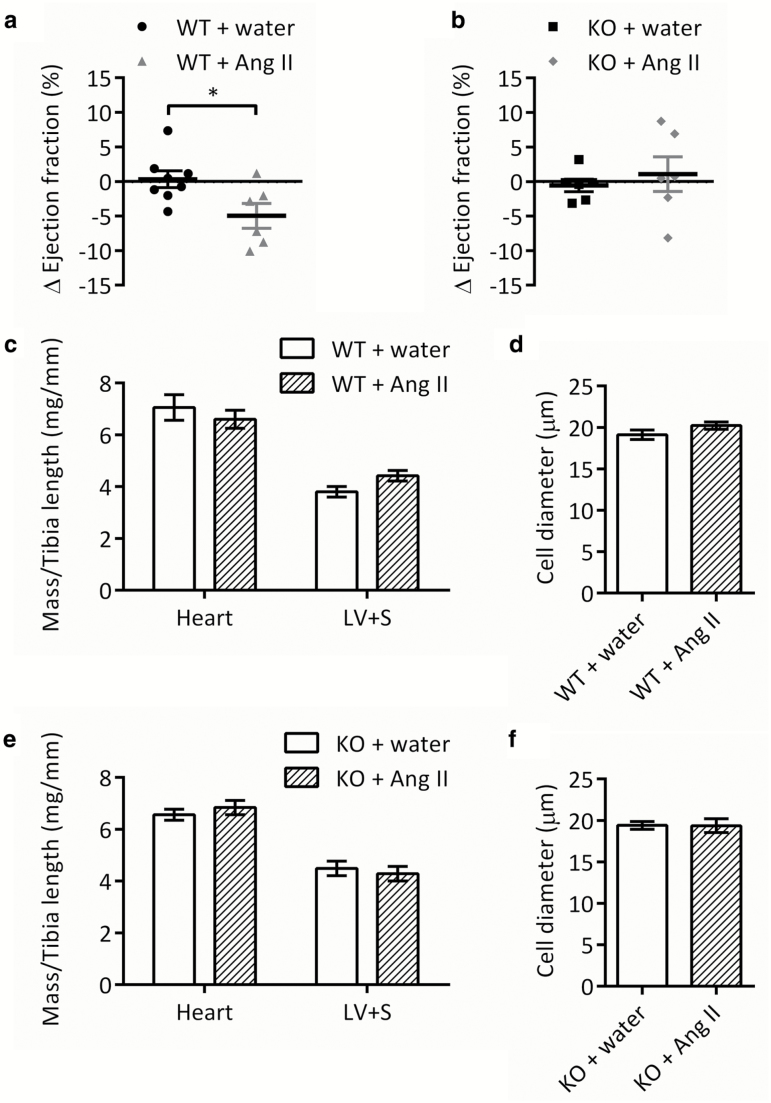
Ejection fraction, cardiac mass, and cardiomyocyte morphology in wild-type and GPR35 KO mice following 2-week Ang II infusion. (**a,b**) Change from baseline at day 14 in ejection fraction calculated from LV dimensions of (a) WT (*n* = 6–8) and (b) GPR35 KO mice (*n* = 6). (**c,e**) Whole heart and LV + S mass of (c) WT and (e) GPR35 KO mice determined at sacrifice and normalized to tibia length; *n* = 7. (**d,f**) Cell diameter in transverse heart sections from (d) WT and (f) GPR35 KO mice quantified by measuring the width of cardiomyocytes orientated on the short axis; *n* = 5. Data are mean ± SEM, compared using 2-tailed unpaired *t* test; no significant differences were found. Abbreviations: Ang II, angiotensin II; GPR35, G protein–coupled receptor 35; KO, knockout; LV, left ventricular; S, septum; WT, wild type.

In wild-type mice, LV wall thickness and diameter were unchanged after either water or Ang II infusion (Table 4); however, in wild-type mice infused with Ang II, FS and EF were significantly reduced on day 14 compared with day 0 (by 6.3 ± 2.2% and 5.7 ± 2.1%, respectively; both *P* < 0.05; Figure 3a and Table 4). The changes in FS and EF in wild-type Ang II-infused mice were significant when compared with those in water-infused mice (both *P* < 0.05; Figure 3a and Table 4).

In GPR35 knockout mice, none of the echocardiographic measurements taken were significantly different on day 14 compared with day 0 (Table 4). The changes in FS and EF were close to 0% in both water- and Ang II-infused GPR35 knockout mice and were not significantly different between groups (Figure 3b and Table 4).

**Table 4.** Echocardiographic parameters in wild-type and GPR35 knockout mice following 2-week Ang II infusion

Measurement	Water	Ang II	*P* value
Wild type			
Δ LV wall thickness, mm	0.12 ± 0.09	0.02 ± 0.07	0.422
Δ LV systolic diameter, mm	0.02 ± 0.10	0.16 ± 0.13	0.422
Δ LV diastolic diameter, mm	0.07 ± 0.17	−0.05 ± 0.17	0.624
Δ Fractional shortening, %	0.41 ± 1.59	−6.26 ± 2.22	0.027*
Δ End systolic volume, µL	0.19 ± 1.82	2.83 ± 2.61	0.406
Δ End diastolic volume, µL	6.32 ± 12.07	−3.72 ± 12.07	0.576
Δ Ejection fraction, %	0.32 ± 1.22	−4.97 ± 1.80	0.027*
GPR35 KO			
Δ LV wall thickness, mm	0.03 ± 0.10	0.09 ± 0.11	0.592
Δ LV systolic diameter, mm	0.07 ± 0.08	−0.22 ± 0.15	0.112
Δ LV diastolic diameter, mm	0.11 ± 0.12	−0.36 ± 0.18	0.057
Δ Fractional shortening, %	−0.85 ± 1.25	1.40 ± 3.30	0.539
Δ End systolic volume, µL	1.37 ± 1.62	−4.39 ± 2.91	0.115
Δ End diastolic volume, µL	9.14 ± 9.17	−25.26 ± 12.89	0.054
Δ Ejection fraction, %	−0.56 ± 0.92	1.07 ± 2.52	0.557

Data are mean ± SEM of change from baseline at day 14 of (wild type: *n* = 6–8; GPR35 KO: *n* = 6). *P < 0.05, compared using 2-tailed unpaired *t* test (water vs. Ang II). Abbreviations: Ang II, angiotensin II; GPR35, G protein–coupled receptor 35; KO, knockout; LV, left ventricular.

Ang II infusion did not significantly affect whole heart or LV mass in either wild-type or GPR35 knockout mice (Figure 3c and e). There was also no detectable induction of hypertrophy at the cellular level, with no significant increase in cardiomyocyte diameter observed in either strain (Figure 3d and f). There were no differences in perivascular or interstitial cardiac fibrosis (Supplementary Figure 1a and b).

**Figure 4.**
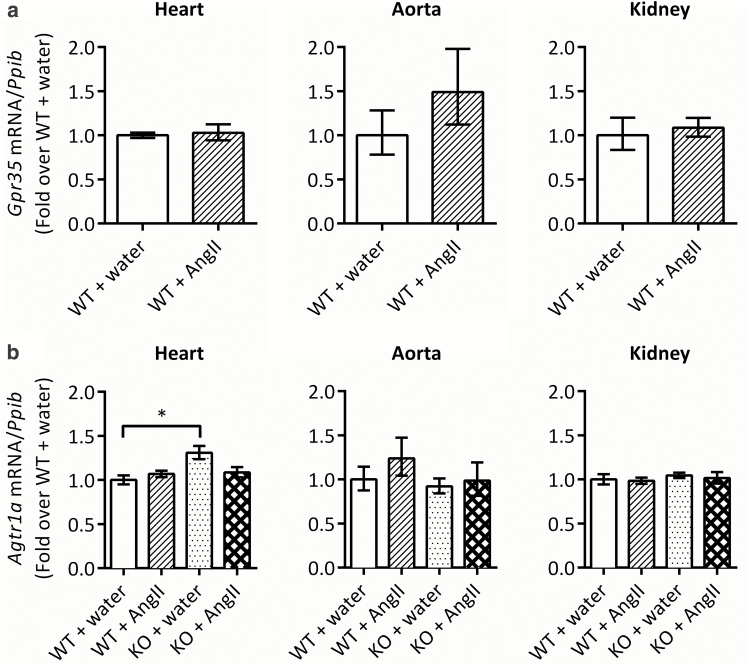
*Gpr35* and *Agtr1a* expression in heart, aorta, and kidney in wild-type and/or GPR35 KO mice following 2-week Ang II infusion. (**a**) *Gpr35* expression relative to housekeeper *Ppib* in wild-type mice. Data are mean ± RQ_max_ and RQ_min_ of *n* = 4; ΔCT values were compared using 2-tailed unpaired *t* test; no significant differences were found. (**b**) *Agtr1a* expression relative to housekeeper *Ppib* in wild-type and GPR35 KO mice. Data are mean ± RQ_max_ and RQ_min_ of *n* = 4; ΔCT values were compared using 1-way ANOVA with Tukey’s multiple comparisons post-test; *P < 0.05. Abbreviations: ANOVA, analysis of variance; Ang II, angiotensin II; GPR35, G protein–coupled receptor 35; KO, knockout; WT, wild type.

*Gpr35* expression in the heart, aorta, and kidney tissue of the wild-type mice infused with Ang II was not significantly different to that of the water-infused mice (Figure 4a). Expression of *Agtr1a* was not significantly different between any 2 groups, except between water-infused wild-type mice and GPR35 knockout mice in the heart (1.3-fold higher expression of *Agtr1a* in GPR35 knockout; Figure 4b). There were no differences in vascular remodeling, renal mass, or renal fibrosis (Supplementary Figures 2 and 3).

## DISCUSSION

The data presented here demonstrate that under physiological conditions, there are no significant differences in hemodynamic parameters, cardiac function, or cardiac morphology in GPR35 knockout mice compared with the wild-type background strain. However, under the pathological conditions of Ang II infusion, GPR35 knockout mice were protected from Ang II–induced increases in blood pressure and impairment of cardiac function.

The finding that GPR35 knockout mice have a normal blood pressure phenotype under basal conditions contrasts with a previous study in such mice, which reported a systolic blood pressure 37.5 mm Hg higher than wild-type littermates.^14^ There are several reasons why these 2 studies might have had such different findings, stemming from the fact that they employed 2 different methods of measuring blood pressure. Min *et al.* used Millar catheterization to record hemodynamic parameters at a single time-point under terminal anesthesia, whereas in this study, blood pressure was monitored continuously *via* telemetry for 7 days in conscious animals. In addition, the age and/or sex of the mice may have influenced the results. As in the majority of cardiovascular studies of animal models not examining sex differences, this study was performed on young adult male mice to avoid potential confounding effects of hormonal variation. It is therefore possible that a high blood pressure phenotype may manifest in female or aged mice and it will be important to study female mice in the future.

LV global systolic function, cardiac mass, cardiomyocyte morphology, cardiac fibrosis, renal mass, renal fibrosis, and vascular remodeling in GPR35 knockout mice were found to be comparable to those in wild-type mice. As GPR35 expression in the heart has been linked to adverse cardiac remodeling, and heterologous overexpression of GPR35 has been shown to drive cardiomyocyte hypertrophy, one might expect GPR35 knockout mice to have altered cardiac mass, structure, or function.^14,15^ However, GPR35 expression in the heart was previously reported following coronary artery ligation or transaortic constriction, or was upregulated in response to hypoxia-inducible factor 1 activation.^15^ This suggests that induction of GPR35 expression depends on the pathophysiological status, which may explain the lack of an abnormal cardiac phenotype in GPR35 knockout mice under physiological conditions.

To investigate the role of GPR35 in the context of cardiovascular disease, we studied the cardiovascular phenotype of the GPR35 knockout mouse in an Ang II infusion hypertension model. GPR35 knockout mice were resistant to the development of Ang II–induced hypertension, suggesting an important role for GPR35 in hypertensive signaling. Gene expression analysis demonstrated that this resistance was not due to *Agtr1a* downregulation in GPR35 knockout mice. There is currently no evidence that GPR35 interacts directly with Ang II, *Agtr1,* or other renin–angiotensin system components. Furthermore, there is very low expression of GPR35 relative to renin–angiotensin system components in key tissues.^23^ Therefore, it is unknown whether GPR35 exerts effects on Ang II–induced hypertension *via* renin–angiotensin system modulation. The loop diuretic drugs bumetanide and furosemide are reported to be human, but not rodent, GPR35 agonists,^24^ highlighting that future studies into GPR35 function may identify novel interactions between GPR35, the renin–angiotensin system, and kidney in blood pressure regulation. Furthermore, since the central nervous system is also integral to blood pressure regulation following Ang II infusion, investigation of GPR35 in the brain would also be interesting in the future. Such studies would be greatly facilitated by availability of selective and potent rodent GPR35 agonists and antagonists.

GPR35 couples selectively to Gα_13_^17,25^ and regulates actin reorganization in both cardiomyocytes and vascular smooth muscle cells.^14–16^ In rodent neonatal and human HL-1 cardiomyocytes, direct overexpression of GPR35 altered actin organization and cell morphology.^15^ Therefore, the cardiovascular effects of GPR35 may be mediated by the Gα_13_/RhoA/ROCK pathway. Although our findings conflict with a previous study in GPR35 knockout mice, existing evidence of pathological roles for Gα_13_/RhoA/ROCK signaling in the cardiovascular system, including prohypertensive and prohypertrophic effects, provide a rationale for why GPR35 knockout mice are protected from cardiovascular disease.^26–28^ More in-depth investigation of the activation of these components in GPR35 knockout models of cardiovascular disease may yield insights into potential mechanisms of Ang II–induced hypertension resistance in GPR35 knockout mice.

An alternative explanation for these findings is that GPR35 interacts with Ang II–mediated blood pressure regulation indirectly through its effects in other tissues. Vascular inflammation is now recognized as an important factor in Ang II–induced hypertension and associated end-organ damage.^29,30^ Previous studies linking GPR35 with inflammation provide another potential mechanism by which GPR35 might contribute to Ang II–induced effects on the cardiovascular system.^3,31,32^ GPR35 expression has been detected in spleen, on lymphocytes and on several myeloid cell types, as well as in vascular endothelial cells.^2,16,32,33^ Studies with GPR35 agonists have suggested possible roles in proinflammatory cytokine release, macrophage recruitment, and leukocyte arrest onto the vascular endothelium.^3,31,32^ Therefore, the apparent requirement for GPR35 in Ang II–induced hypertension could be due to a function that contributes to vascular inflammation. Furthermore, recent evidence suggests macrophages originating in the spleen, where GPR35 is highly expressed,^2^ may contribute to Ang II–induced cardiac fibrosis and hypertension.^34^ A role for GPR35 in the prohypertensive immune response is an interesting possibility that has not previously been explored, and future studies into the effects of GPR35 on markers of vascular inflammation and release of immune cells from the spleen in models of hypertension could yield further insight.

In conclusion, the findings presented here suggest that although GPR35 does not appear to influence physiological cardiovascular regulation, it plays an important pathological role in the development of Ang II–induced hypertension and impaired cardiac function, and therefore may contribute to cardiovascular disease. Although the mechanism by which inactivation of GPR35 attenuates Ang II–induced hypertension remains unclear, this suggests that targeting GPR35 could be a novel target in the therapeutic control of blood pressure, most obviously through the use of GPR35 antagonists. The lack of antagonists with affinity for rodent GPR35 orthologues will make investigating the therapeutic potential of GPR35 antagonism challenging. Ongoing screening efforts (supported by novel bioinformatic approaches for ligand identification as well as an improved understanding of the mechanisms of species selectivity) may yet yield equipotent GPR35 antagonists that will enable such studies.^8,35^ It is hoped that future studies that assess the effects of GPR35 antagonism, along with a better understanding of the mechanisms involved in these effects, will reveal its true potential as a pharmacological target in cardiovascular disease.

## SUPPLEMENTARY DATA

Supplementary data are available at *American Journal of Hypertension* online.

Supplementary Information

## DISCLOSURE

The authors declared no conflict of interest.
