# Supplementary material for: The Orphan Receptor GPR35 Contributes to Angiotensin II–Induced Hypertension and Cardiac Dysfunction in Mice
Source: Am J Hypertens. 2018 May 31;31(9):1049–58. doi: 10.1093/ajh/hpy073 (PMC6077831; doi:10.1093/ajh/hpy073)
Supplement: Supplementary Information [file hpy073_suppl_supplementary-information.docx]

**The orphan receptor GPR35 contributes to angiotensin II-induced hypertension and cardiac dysfunction in mice**

# Supplementary Information

# Supplementary Methods

## Picrosirius red staining

Picrosirius red was used to stain collagen fibres in heart and kidney tissue as a measure of cardiac and renal fibrosis. Following deparaffinisation and dehydration, 3 μm heart (left ventricle + septum) and kidney sections were incubated for 5 min in distilled water. Nuclei were stained by incubating in Weigert’s hematoxylin (Sigma-Aldrich) for 10 min, then sections were washed in running tap water for 10 min. Sections were stained with picrosirius red (0.1% (w/v) sirius red in saturated picric acid solution (both Sigma-Aldrich) for 90 min. Sections were washed 2 x 3 min in acidified water (0.01 N HCl), then vigorously shaken to remove water before dehydrating in 100% ethanol for 3 min. Sections were cleared in Histoclear for 2 x 7 min, mounted in DPX mountant and dried overnight at room temperature. Images were taken with a x40 objective on an Olympus BX41 microscope using a QImaging Go-3 camera. The experimenter was blinded to treatment groups during imaging and analysis. For hearts, six fields of view were taken. For kidneys, eight fields of view (four interstitial and four perivascular) were taken. Fibrosis was quantified by measuring the proportion of red pixels using Image-Pro Plus software (Media Cybernetics).

## Hematoxylin and eosin (H&E) staining

Hematoxylin and eosin was used to stain nuclei and cytoplasm in order to visualise the gross morphology of blood vessels. Following deparaffinisation and dehydration, 5 μm aorta or carotid artery sections were incubated for 5 min in distilled water. Sections were stained with Harris hematoxylin (CellPath) for 2 min, then washed in running tap water for 5 min. Sections were incubated in 70% (v/v) ethanol for 1 min, then stained with Eosin Y (CellPath) for 3 min. Sections were washed in 95% (v/v) ethanol for 2 x 30 sec, then dehydrated in 100% ethanol for 2 x 5 min. Sections were cleared in Histoclear for 2 x 5 min, mounted in DPX mountant and dried overnight at room temperature. Images showing the whole vessel were taken with a x4 (aortas) or x10 (carotid arteries) objective on an Olympus BX41 microscope using a QImaging Go-3 camera. The experimenter was blinded to treatment groups during imaging and analysis. Total and lumen areas were measured using ImageJ software and used to calculate medial area (total area − lumen area) and wall-to-lumen ratio (total area − lumen area/lumen area).

# Supplementary Figures


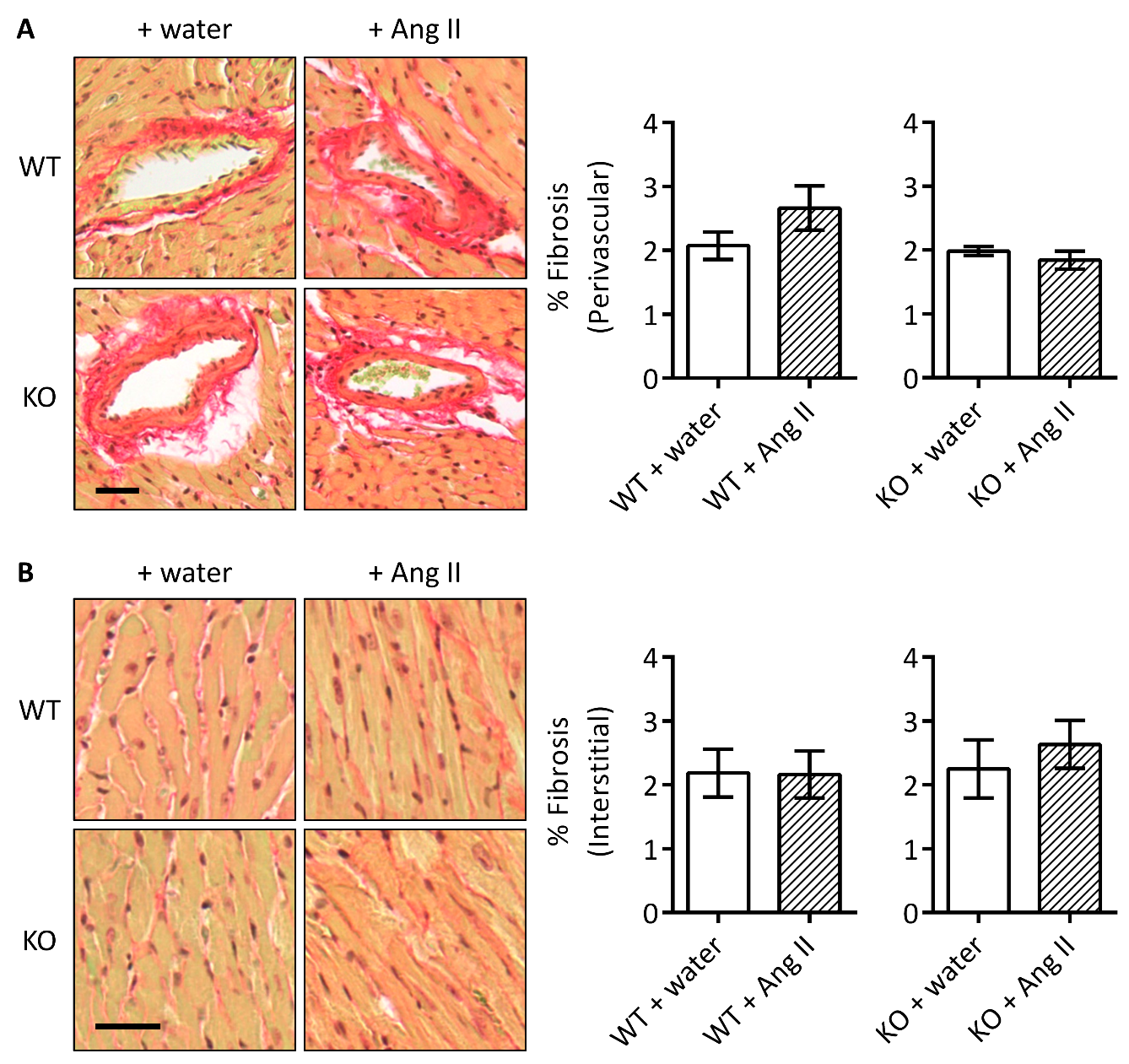


**Supplementary Figure 1** Cardiac fibrosis in wild type and GPR35 knockout mice following two-week Ang II infusion.

Transverse heart sections were stained with picrosirius red to visualise collagen (red staining; scale bar 30 μm). Images of (**A**) perivascular and (**B**) interstitial regions were taken, and percent fibrosis was quantified as percentage of red pixels; n = 7 (wild type + water and KO + Ang II), n = 3 (wild type + Ang II) or n = 4 (KO + water). Data are mean ± SEM, compared using two-tailed unpaired t test; no significant differences were found.

Ang II, angiotensin II; GPR35, G protein-coupled receptor 35; KO, knockout; WT, wild type.


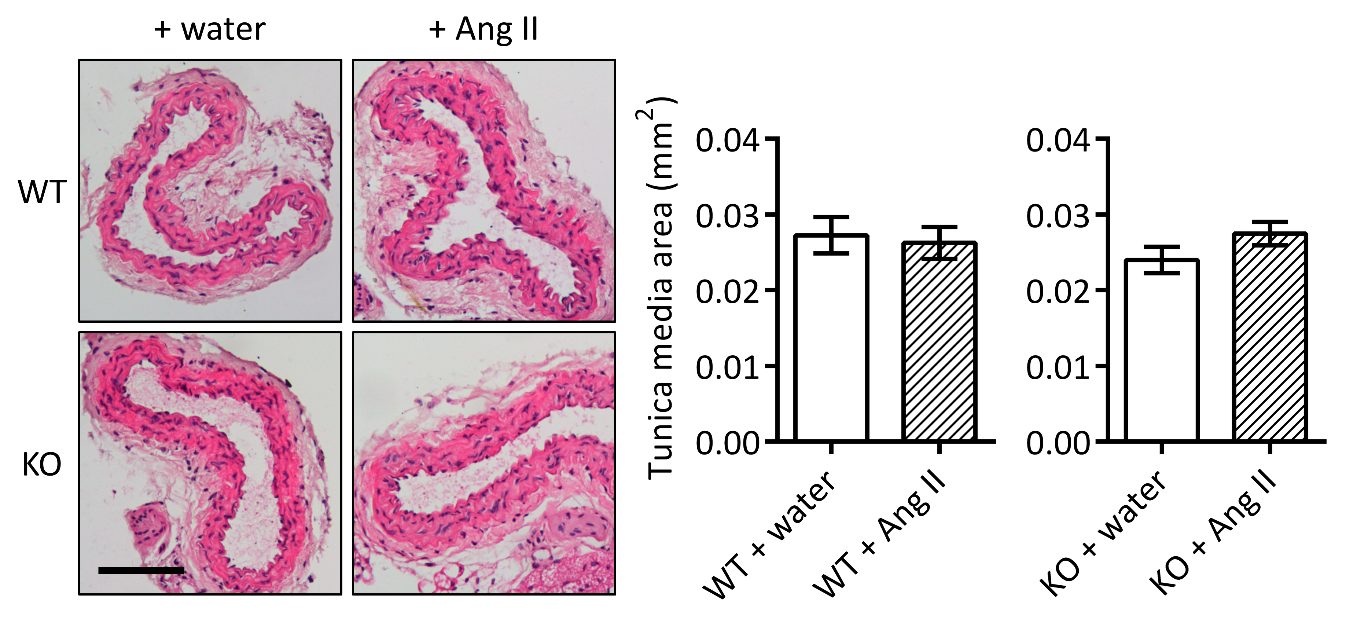
 **Supplementary Figure 2** Vascular morphology in wild type and GPR35 knockout mice following two-week Ang II infusion.

Transverse carotid artery sections were stained with hematoxylin and eosin to visualise vascular morphology, and tunica media area was quantified by measuring the smooth muscle layer (bright pink staining; scale bar 100 μm); n = 4. Data are mean ± SEM, compared using two-tailed unpaired t test; no significant differences were found.

Ang II, angiotensin II; GPR35, G protein-coupled receptor 35; KO, knockout; WT, wild type.


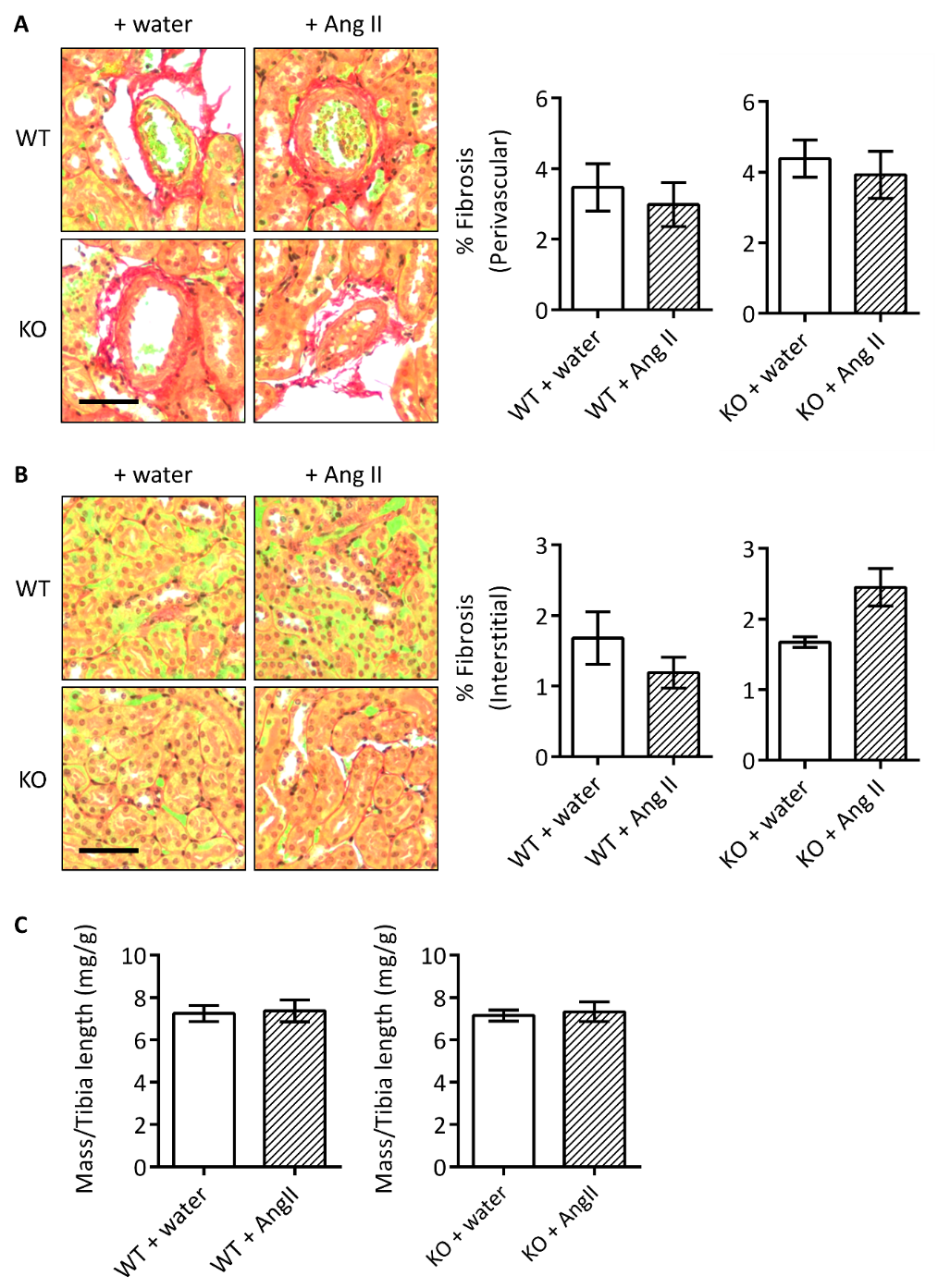


**Supplementary Figure 3** Renal mass and morphology in wild type and GPR35 knockout mice following two-week Ang II infusion.

(**A,B**) Transverse heart sections were stained with picrosirius red to visualise collagen (red staining; scale bar 50 μm). Images of (**A**) perivascular and (**B**) interstitial regions were taken, and percent fibrosis was quantified as percentage of red pixels; n = 4 (wild type + water, wild type + Ang II and KO + Ang II) or n = 3 (KO + water). (**C**) Average kidney mass was determined at sacrifice and normalised to tibia length; n = 7 (wild type + water and KO + Ang II) or n = 6 (wild type + Ang II and KO + water). Data are mean ± SEM, compared using two-tailed unpaired t test; no significant differences were found. Ang II, angiotensin II; GPR35, G protein-coupled receptor 35; KO, knockout; WT, wild type.
